# Preconception genetic carrier screening: use and problems

**DOI:** 10.1080/03009734.2016.1230158

**Published:** 2016-09-22

**Authors:** Viroj Wiwanitkit

**Affiliations:** Surindra Rajabhat University, Surin Province, Thailand

The recent publications on preconception health and care are very interesting (1). Indeed, the ethical issues are important concerns. The published article by Kihlbom focused upon preconception genetic screening with expanded panels, but it is only for one trait (2). I would like to share the practice in my country on the screening of complex endemic genetic problems. In Thailand, genetic carrier screening for preconception thalassemia and hemoglobin disorders has been used for a long time, and it has become accepted as an important policy for management of these endemic hemoglobin disorders (3). There are no problems as regards acceptance amongst the local people. Key factors might be the campaign for health education concerning the importance and seriousness of the disease and making people aware of the problems of the affected families in the community (4).
